# A Comparison of Parenteral and Per-Oral Antibiotic Usage in Pyogenic Flexor Tenosynovitis: A Retrospective Study

**DOI:** 10.7759/cureus.32825

**Published:** 2022-12-22

**Authors:** Kaela Frizzell, Elkin Galvis, Laxminarayan Bhandari

**Affiliations:** 1 Hand Surgery, Christine M. Kleinert Institute of Hand and Microsurgery, Louisville, USA; 2 Hand Surgery, Kleinert Kutz and Associates, University of Louisville, Louisville, USA

**Keywords:** intravenous antibiotic, digital amputation, surgical drainage, antibiotics therapy, septic tenosynovitis

## Abstract

Introduction

Pyogenic flexor tenosynovitis (PFT) is a common hand infection that can cause significant morbidity. Although treatment involves surgical debridement and inpatient intravenous (IV) antibiotics, there is a paucity of literature guiding antibiotic use. This study aims to determine if the use of postoperative outpatient oral antibiotics leads to poor outcomes compared to IV antibiotics given in an institutional setting.

Methods

A retrospective review of 110 patients treated post-operatively with either outpatient oral or inpatient IV antibiotics at our institution from 2016-2019 was performed. All patients underwent surgical debridement. Primary outcomes analyzed included readmission, repeat surgery, and amputation. Clinical parameters including age, diabetes, smoking, duration of symptoms, involvement of surrounding structures (felon, dorsal abscess, osteomyelitis, septic arthritis), culture growth, Michon classification, and duration of antibiotics were analyzed as possible risk factors for poor outcome. The level of evidence of this study is Level 3 Retrospective Cohort Study.

Results

Seventy-five patients were treated with outpatient oral antibiotics and 35 patients were treated with inpatient IV antibiotics. The oral antibiotics group received antibiotics for 13.1 +/- 9.9 days compared to 18.1 +/-10.4 days in the IV antibiotic group. Patients in the oral antibiotic group had a significantly shorter length of hospitalization at 0.6 +/-1.8 days compared to 3.6 +/-1.8 days in the IV antibiotic group. The readmission rate for the oral antibiotic group was 10.7% compared to 5.7% in the IV antibiotic group. This difference was not statistically significant except in patients who had involvement in surrounding structures. There was no significant difference in repeat surgeries or amputations between the groups.

Conclusions

The use of outpatient oral antibiotics after surgical debridement for PFT does not significantly increase rates of readmission, repeat surgery, or amputation, except in cases with the involvement of surrounding structures. On subgroup analysis, anaerobic infection and diabetes were significantly associated with amputations. Post-operative oral antibiotics and immediate discharge may be considered for PFT after adequate surgical debridement with close outpatient follow-up in the absence of surrounding structure involvement and diabetes.

## Introduction

Pyogenic flexor tenosynovitis (PFT) was first described by Allen Kanavel over one hundred years ago as an infection of the flexor tendon sheath that can cause significant disability if not treated appropriately [1]. The importance of surgical treatment of flexor tenosynovitis was first described prior to the advent of penicillin. Grinnell described the surgical treatment of 125 cases with over one-third poor results and an 8% amputation rate prior to antibiotic use [2]. Most literature regarding this topic in the past eighty years was focused on surgical techniques alone, despite the significant effect of antibiotics on the outcomes [3-8]. After the introduction of antibiotics, the results improved, however, ambiguity still persists regarding antibiotic recommendations [3,9-10]. Recent review articles on flexor tenosynovitis have given no recommendations for the route of administration or duration of postoperative antibiotics [3,11-12]. 

The primary goal of this study was to provide guidance regarding postoperative antibiotics for PFT. We examined the outcomes of patients treated for PFT with surgical treatment and post-operative oral antibiotics or intravenous (IV) antibiotics. Primary outcomes included readmission, re-operation, and amputation. We hypothesized that the use of oral antibiotics after operative treatment for PFT would not result in increased readmissions, re-operations, or amputations compared to IV antibiotics.

## Materials and methods

After obtaining Institutional Review Board (IRB) approval at our institution (University of Louisville Institutional Review Board, approval number 19.1065), we identified patients with the International Classification for Disease 10 (ICD10) diagnosis codes for flexor tenosynovitis (M65.041, M65.042, M65.049, M65.841, M65.842, M65.849) at our institution from 2016-2019. All charts were reviewed. One hundred and thirty-four patients treated operatively for PFT were identified. The diagnosis of PFT was made clinically by the presence of one or more of Kanavel’s signs: fusiform swelling, pain with passive extension, tenderness to palpation of the flexor sheath, and/or flexed posturing.

Multiple surgeons at our institution including attendings and fellows performed the surgery. The selection of oral or IV antibiotics was as per the surgeon's preference. Postoperatively, patients in the oral group were discharged immediately after operative intervention. Patients usually received 7-14 days of oral antibiotics, most often amoxicillin + clavulanic acid or trimethoprim + sulfamethoxazole. Another antibiotic prescription was given at follow-up if there were still signs of infection not necessitating operative intervention. Patients in the IV group were admitted for post-operative IV antibiotics.

Patients with less than one week of outpatient follow-up and intra-operative intervention for extension of infection into the carpal tunnel, hypothenar, or thenar bursa were excluded.

We examined age, diabetes, smoking, duration of symptoms, involvement of surrounding structures (felon, dorsal abscess, osteomyelitis, septic arthritis), culture growth, Michon classification, and duration and route of antibiotics as possible risk factors for poor outcome (Table 1) [13].

**Table 1:** Michon Classification of Pyogenic Flexor Tenosynovitis

Michon Stages	Findings
Stage 1	Increased fluid in sheath, primarily serous exudate
Stage 2	Cloudy or purulent fluid, granulomatous synovium
Stage 3	Septic necrosis of tendon, tendon sheath or pulleys

Statistical analysis was performed with a Fisher's exact test for the association between categorical data and a Mantel-Haenszel to test the association between binary data.

## Results

We identified 138 patients treated for PFT from 2016-2019; 28 patients were excluded for not having any follow-up. Seventy-five patients treated with oral antibiotics and 35 patients treated with IV antibiotics were included. Fisher's Exact test was used to determine power analysis for each test, and the power was found to be between 80-90 indicating good power for the tests. The average age of patients receiving oral antibiotics was 45.9 years (range 16-86) compared to 48.3 years (range 24-83) in the IV group. 

The incidence of diabetes was 7 out of 35 (20%) in the IV group and 7 out of 75 (9.3 %) in the oral group. This was not found to be statistically significant. The IV antibiotic group had significantly more patients (11 out of 35 or 31.4%) with involvement of surrounding structures such as a dorsal abscess, felon, osteomyelitis, or septic arthritis than the oral group (8 out of 75 or 10.7%, p=0.01). In the IV antibiotics group, 27 out of 35 (80%) patients had a growth on culture compared to 43 out of 75 (57.3%) patients in the oral group (p=0.06). Forty out of 75 (54%) patients in the oral antibiotics group had either purulence or tendon necrosis compared to 20 out of 35 (57%) in the IV group (p=0.08) (Table 2).

**Table 2:** Demographics and Group Characteristics *Dorsal abscess, felon, septic arthritis, osteomyelitis #Includes hospitalization time if readmitted

Parameter	Oral Antibiotics (N=75)	IV Antibiotics (N=35)	p-value
Age	45.87 (range 16-86)	48.34 (range 24-83)	0.96
Smokers	37 (49.3%)	15 (42.9%)	0.55
Diabetes	(7) (9.3%)	7 (20%) (7)	0.13
Duration of symptoms (days)	3.33 ( range 1-21)	3.94 (range 1-14)	0.08
Involvement of surrounding structures*	8 (10.7%)	11 (31.4%) )	0.01
Culture growth	43 (57.3%)	27 (80%)	0.06
Michon classification			0.08
Stage I	35 (46.7%)	(15) 42.9%	
Stage II	38 (50.7%)	(15) 42.9%	
Stage III	2 (2.7%)	(5) 14.3%	
Total duration of antibiotics (days)	13.1 +/- 9.9	18.1 +/-10.4	0.09
Total length of hospitalization (days)#	0.6 +/-1.8	3.6 +/-1.8	<0.01
Follow-up (weeks)	13.2 +/-22.2	19.4 +/-29.9	0.19

The oral antibiotics group received antibiotics for 13.1 +/- 9.9 days compared to 18.1 +/-10.4 days in the IV antibiotic group (p=0.09). Patients in the oral antibiotic group had a significantly shorter length of hospitalization at 0.6 days compared to 3.6 days in the IV antibiotic group (p<0.01). The length of hospitalization included time if readmitted. 

Readmission rate

The readmission rate for the oral antibiotic group was 10.7% (8 out of 75) compared to 5.7% (2 out of 35) in the IV antibiotic group (p=0.5). There was no significant difference in readmission rates except in patients who had involvement in surrounding structures. In the oral antibiotic group, 50% (4 out of 8) of patients with involvement of surrounding structures were readmitted. None of the patients in the IV groups with involvement of surrounding structures were readmitted (p=0.04) (Table 3).

**Table 3:** Outcomes: Oral vs. IV Antibiotics *All revision amputations

Parameters	Oral Antibiotics (N=75)	IV Antibiotics (N=35)	p-value
Readmission	8 (10.7%)	2 (5.7%)	0.5
Involvement of surrounding structures	4 (50%)	0	0.04
Repeat Surgery for Infection	14 (18.7%)	8 (22.9%)	0.6
Amputation	1 (1.3%)	6 (17.1%)	<0.01
Amputation after index procedure*	0	4 (11.4%)	<0.01

Repeat surgery

There was no significant difference in repeat surgeries between the groups (14 out of 75, or 18.7%, in the oral group vs. 8 out of 35, or 22.9%, in the IV group, p=0.6). Seventy-three percent of repeat surgeries were performed within seven days postoperatively.

Amputation

In our cohort, there was an overall amputation rate of 6.4% (7 out of 110). The IV antibiotic group had a significantly higher rate of amputation (6 out of 35, 17.1%) during the index procedure and subsequent surgeries (p<0.01) than the oral antibiotic group (1 out of 75, 1.3%). There was a significant increase in amputations in the IV antibiotics group in patients with less than three days of symptoms (p<0.01) and Michon classification stage 2 (p<0.01). 

Type of bacteria

The following bacteria grew in cultures - Methicillin-sensitive Staph Aureus, Methicillin Resistant Staph Aureus, Group A Streptococcus, Group B Streptococcus, Pasturella, and anaerobes. However, the only significance was noted with anaerobes. On further subgroup analysis, no types of bacteria were found to be significant in predicting the odds of being readmitted or of repeat surgery (p>0.4). However, anaerobe had a significant odds ratio of predicting amputation (p=0.012).

Diabetic status

When controlling for which post-operative antibiotic regimen, there was a significant association between diabetes and amputation (p=0.003).

Type of surgery

There was no statistical significance found between closed and open surgery (p>0.05) and readmission, repeat surgery, or amputation. 

## Discussion

The present study sought to evaluate postoperative antibiotic regimens for PFT. Overall, the use of oral antibiotics did not significantly increase the rates of readmission, repeat surgery, or amputation compared to IV antibiotics in the current study. The only exception was in patients with involvement of surrounding structures who had increased rates of readmission. 

Amputation is a devastating result of pyogenic flexor tenosynovitis. In our study, there was a higher rate of amputations in the IV group both at index procedures and during secondary procedures. IV antibiotics were likely chosen due to the severity of infection at presentation in these cases; therefore there is an inherent bias. However, it is important to note there were no amputations after the first surgery with oral antibiotics.

To our knowledge, there have not been any similar studies comparing postoperative oral and IV antibiotics in PFT. Most recent literature focuses on the results of operative intervention, specifically amputation and range of motion. Pang et al. examined factors contributing to poor outcomes in PFT. They found increased amputation rates and decreased total active motion in patients with age greater than 43, diabetes, peripheral vascular disease, chronic kidney disease, more than one bacteria type, subcutaneous purulence, and ischemic changes at presentation [14]. Our study has similar findings. We found diabetic status (p=0.003) and anaerobic infections (p=0.012) to have a significant association with amputation. 

In our cohort, 7 out of 110 patients underwent amputation at index procedure resulting in an overall amputation rate of 6.4%, Out of these seven patients, four underwent additional amputation as a secondary procedure. All the secondary procedures were in the IV group. In Pang et al.'s cohort, amputation was performed at a mean of nine days (range 5-14 days) after admission and three days (range 2-6 days) after initial debridement. Our surgeons performed amputation within twenty-four hours of presentation, which is considerably more aggressive than the nine days in Pang et al.’s cohort; although in their series of 75 patients, there was an overall amputation rate of 17%. Dailiana et al. found that delayed treatment leads to worse outcomes [15]. Aggressive early debridement likely contributed to overall lower rates of amputation and successful outcomes with oral antibiotics alone. Hohendorff et al. had similar success in their series of 22 patients with a short course of IV antibiotics transitioned to oral antibiotics. They suggested a surgically treated infection no longer needs prolonged postoperative antibiotic treatment [16].

A recent study by DiPasquale et al. described good results with non-operative treatment with IV antibiotics alone; although the average hospitalization was five days, which can be expensive and time-consuming for the patients. The authors recommend against non-operative treatment in cases with purulence in the flexor tendon sheaths, which can be difficult to determine clinically [17].

The use of oral antibiotics theoretically decreases overall cost, but further studies would be necessary to confirm this. Many authors have recommended a trial of inpatient IV antibiotics at least twenty-four hours prior to operative intervention; however, the cost and inconvenience to the patient should be given due consideration [3,9,11,12,17]. A prospective randomized controlled study would eliminate the inherent treatment bias of selecting IV antibiotics for more severe infections. However, treating such infections with only oral antibiotics may be clinically inappropriate.

The current study is not without limitations. The data in the charts were limited and we cannot make any assessment of patient satisfaction with their antibiotic regimen or range of motion as this was inconsistently charted. Second, multiple surgeons performed the surgeries and, notably, 64% of the patients requiring readmission or repeat surgery in the oral group had surgery done by a fellow in the minor treatment room. As the experience levels of surgeons were not standard, there is a chance of inadequate debridement at initial surgery that might have confounded the outcomes.

## Conclusions

In conclusion, surgical drainage followed by oral antibiotics may be considered in the appropriate patient with flexor tenosynovitis. In patients with involvement of surrounding structures such as a felon, septic arthritis, osteomyelitis, or dorsal abscess, IV antibiotics are warranted. A thorough operative debridement and close follow-up are still needed. Diabetes and anaerobic infection have a significant association with amputation.
